# Performance-based readability testing of participant information for a Phase 3 IVF trial

**DOI:** 10.1186/1745-6215-10-79

**Published:** 2009-09-01

**Authors:** Peter Knapp, DK Raynor, Jonathan Silcock, Brian Parkinson

**Affiliations:** 1 School of Healthcare, University of Leeds, Leeds, UK; 2 Making Sense Design, Sheffield, UK

## Abstract

**Background:**

Studies suggest that the process of patient consent to clinical trials is sub-optimal. Participant information sheets are important but can be technical and lengthy documents. Performance-based readability testing is an established means of assessing patient information, and this study aimed to test its application to participant information for a Phase 3 trial.

**Methods:**

An independent groups design was used to study the User Testing performance of the participant information sheet from the Phase 3 'Poor Responders' trial of In Vitro Fertilisation (IVF). 20 members of the public were asked to read it, then find and demonstrate understanding of 21 key aspects of the trial. The participant information sheet was then re-written and re-designed, and tested on 20 members of the public, using the same 21 item questionnaire.

**Results:**

The original participant information sheet performed well in some places. Participants could not find some answers and some of the found information was not understood. In total there were 30 instances of information being not found or not understood. Answers to three questions were found but not understood by many of the participants, these related to aspects of the drug timing, Follicle Stimulating Hormone and compensation. Only two of the 20 participants could find and show understanding of all question items when using the original sheet. The revised sheet performed generally better, with 17 instances of information being not found or not understood, although the number of 'not found' items increased. Half of the 20 participants could find and show understanding of all question items when using the revised sheet. When asked to compare the versions of the sheet, almost all participants preferred the revised version.

**Conclusion:**

The original participant information sheet may not have enabled patients fully to give valid consent. Participants seeing the revised sheet were better able to understand the trial. Those who write information for trial participants should take account of good practice in information design. Performance-based User Testing may be a useful method to indicate strengths and weaknesses in trial information.

## Background

Over the past decade writers have expressed concern about the process of consent to clinical trials and the levels of knowledge about the trial that participants have. Three surveys of patients' understanding at the end of a trial have found sub-optimal comprehension, such as one fifth not knowing the name of the medicine being tested [1] and 30-40% patients not knowing that they could withdraw from the trial at any time [2,3]. A study of healthy trial volunteers reported that almost all participants could recall the procedure being researched, but only a small minority could recall drugs used in the trial or potential side effects [4]. This finding of variable knowledge is confirmed by a systematic review of communication and informed consent in cancer trials. [5] It suggested that aspects such as risks and benefits associated with the tested medicine and the right to withdraw consent, were understood less well than others. The review concluded that "patients do not appear to be adequately informed of the aims of (Phase 1) trials" (p.304).

There can be many reasons for a lack of knowledge: information sheets may be too long or too complex [4,6], or the recruiting clinician might not check patient understanding [7]. Furthermore, levels of knowledge may vary according to participant characteristics. For example, understanding of trials was shown to be poorer among older patients and those with fewer years in education [8].

Finding a low level of knowledge of aspects of the trial might question the extent to which consent was valid or informed. It might also impact on the running of the trial. One study found higher levels of "later decisional regret" among trial participants who thought they had been less informed about their decision to participate [9].

The concern about participant understanding was heightened as a result of the serious adverse events among healthy volunteers in the TGN1412 Phase I trial in 2006. The Expert Scientific Group, formed as a result of the incident, reported that the process of informed consent and clarity of participant information were "extremely important" and should be "taken up as a high priority and considered in detail" [10]. The Royal Statistical Society report criticised the TGN1412 information sheets, particularly for the way that the treatment allocation schedule was explained, and the use of difficult, technical words [6].

The participant information sheet performs different purposes before and after consent is taken. Pre-consent the sheet is used, along with spoken information from the recruiting clinician, to explain the trial: its purpose; what consent means; the tested medicine; and, the patient's responsibilities. The sheet can also help the patient to formulate questions.

Post-consent the sheet's functions are slightly different. It serves to remind the patient of what they have agreed to, and it can also act as a memory aid on particular aspects of the trial. After consent the patient needs to be able to understand the sheet without access to a clinician. This last point emphasises the need for the sheet to be written and presented such that participants can find and understand information. The role of the participant information sheet would seem to be particularly important in studies of complex interventions, such as in In Vitro Fertilisation (IVF), when different medicines are administered for varying lengths of time throughout.

One method for assessing readability is the use of formulae [11-13]. These tests can be used to assess any written document, including trial information. One review of information sheets by readability formulae suggests that most require too high a standard of literacy in readers [14]. Formulae are often used by US Institutional Review Boards, who require that sheets score at a certain level of readability, before approving a trial [15].

Readability formulae can be easily used, and the generated score largely depends on word and sentence length. A major weakness is that they cannot assess the meaning of information - the phrase "*injection by given be will medicine the*" will attain the same readability score as "*the medicine will be given by injection*" - and, perhaps more importantly, they cannot indicate how the information will perform. For example, they cannot show whether patients will understand safety issues, or procedures, or randomisation. The limited value of formulae has led to recommendations to test participant information sheets by their performance [16,17]. Recent performance-based testing of a Phase I trial sheet found significant readability problems. The study also reported an improved level of performance after the sheet had been re-worded and re-designed [18].

Testing the performance of patient information is relatively recent [19] and it has gained impetus since European law has required medicine manufacturers to test the patient information leaflets supplied with every licensed medicine [20]. Market authorisation for a new drug will not be granted until a successful, documented test has been conducted. As a consequence, since 2005 medicine leaflets across Europe, including several thousand in the UK, have been tested for readability using a performance-based method. In almost all cases the method used has been 'User Testing', as described in guidance documents [21]. User Testing involves potential medicine users reading the information, and then being asked to find and show understanding of 12-15 items of information. (The term 'User Testing' can be misunderstood - it is the users who are testing the information, rather than the users being tested.) Participants are usually potential users of the information, rather than those currently taking the medicine, whose prior knowledge might bias results. User Testing is intended to be iterative, with the information being revised after rounds of 10 participants, to remedy any identified flaws. The EU legislation requires that the final version of the leaflet should have been tested on two rounds of 10 people, and that each can be found and understood by at least 8 of every 10 people [20,21]. Since the 1990s User Testing has been applied to medicine leaflets in Australia [22].

Several studies have examined the effect of making changes to trial participant information sheets. Shortening the information about an asthma trial led to greater understanding of a number of aspects (including randomisation, duration, and benefits) [23]. Re-writing participant information into an 'easy-to-read' format led to greater satisfaction and less anxiety among participants [24], although understanding of the information was not assessed. However, one study found no effect of offering different explanations of aspects of trials, such as equipoise and random allocation [25].

This paper reports the User Testing method in relation to the participant information used in the 'Poor Responders' IVF trial [26], a trial with relatively complex interventions. It assesses whether people could find and understand key pieces of information related to the trial. The test results are then used to inform the revision of the participant information sheet.

## Methods

### Design

An independent groups design was used, with each participant seeing only one version of the information.

### Participants

40 healthy members of the public were recruited via newspaper advertising and promotional flyers distributed to houses and businesses in the local area. They had been recruited to take part in readability studies. We also approached fertility and IVF support groups in the area, in order to recruit women with personal experience of IVF. Participants were adult women aged under 45, as in the actual 'Poor Responders' IVF trial. We excluded women who had taken part in any medicine trial or readability testing study in the previous 6 months. In addition we ensured that each round of 10 participants had a similar profile in terms of likely influences on testing: age, education and occupation. We also ensured that each round contained a similar number of women who had themselves received IVF treatment, in case their experience influenced understanding of the trial information.

### Tested Materials

**Figure 1:**
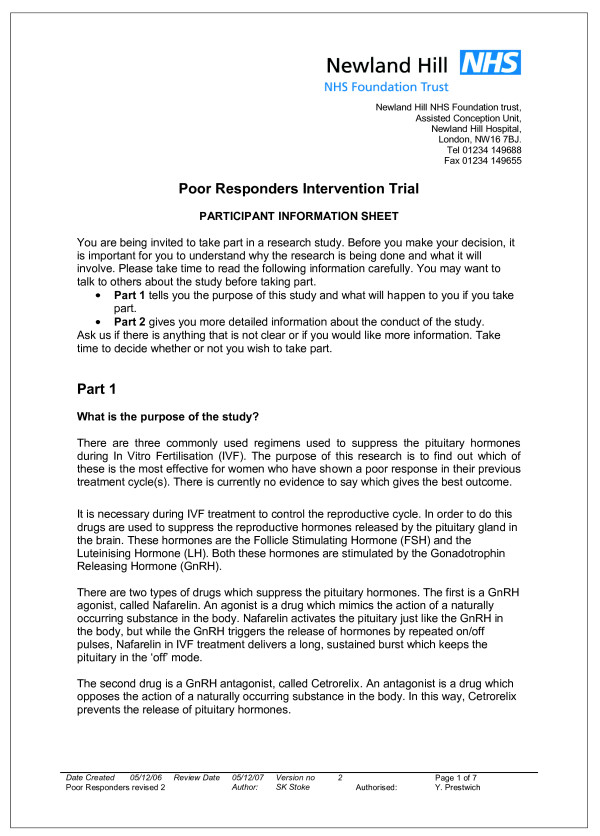
**Front page of the original participant information sheet**.

**Figure 2:**
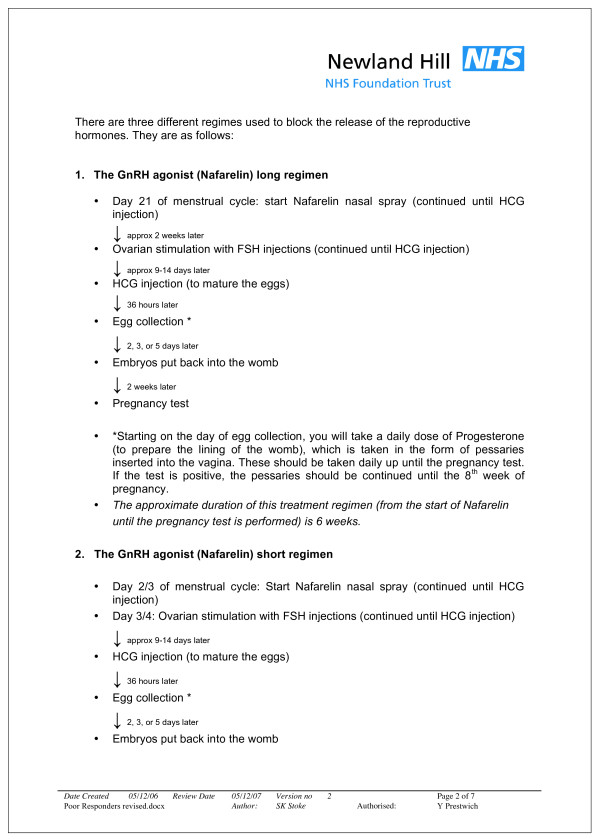
**Treatment timeline as described in the original participant information sheet**.

**Figure 3:**
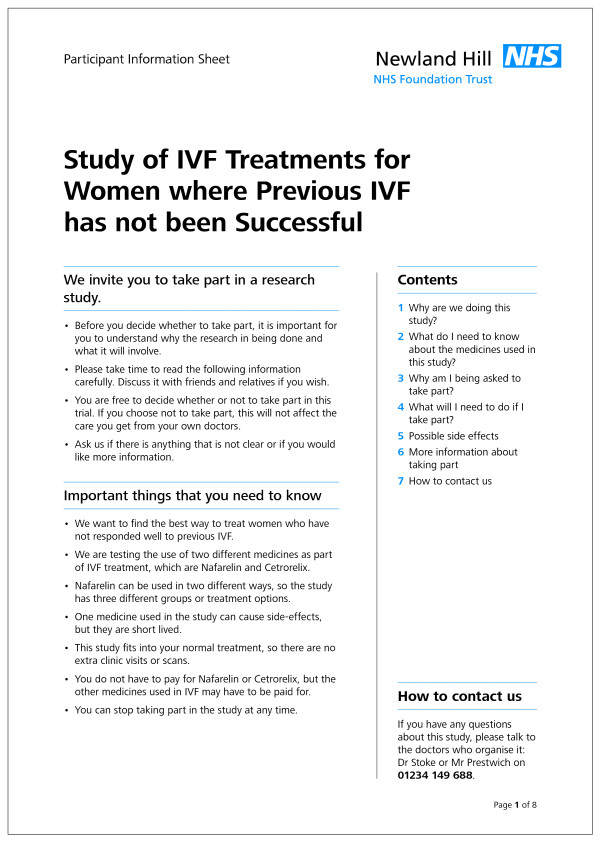
**Front page of the revised participant information sheet**.

**Figure 4:**
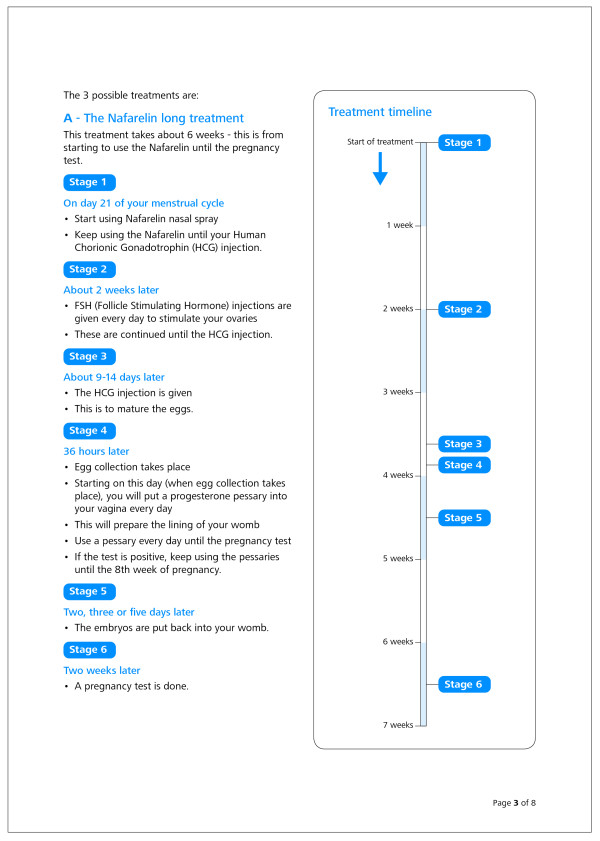
**Treatment timeline as described in the revised participant information sheet**.

(i) The original 'Poor Responders' trial participant information sheet, comprising 7 pages of single-sided A4 paper (see Figures 1 and 2 for example sections). This was obtained from the article reporting the trial methods [26]. We contacted the authors to inform them that we intended to test the trial information sheet for readability, and asked them to confirm how it was presented to potential participants. Before testing all content identifying individuals or organisations was replaced with pseudonyms.

(ii) A revised version of the 'Poor Responders' trial sheet, retaining its meaning but with revised format, appearance and wording. See below for further detail on this revision process (and see Figures 3 and 4 for example sections).

### Outcomes

Participants' ability to find 21 key points of information in the sheets, and then convey their understanding of each of those points (see Table 1). The 21 items were drawn from four categories of information, being those that would apply to trials of any phase:

**Table 1:** Testing results of the original and revised versions of the participant information sheets

	**Rounds 1 & 2: Original sheets **(n = 20)		**Rounds 3 & 4: Revised sheets **(n = 20)	
	
	**Found (of which, found with difficulty)**	**Understood**	**Found (of which, found with difficulty)**	**Understood**
**Nature and purpose of the trial**				

**Q1 **Who is funding this trial?	20 (0)	20	20 (0)	20

**Q2 **What is the purpose of this trial?	20 (2)	18	20 (1)	17

**Process and meaning of consent**				

**Q4 **What would happen to any information collected from you as part of the trial?	20 (4)	17	20 (2)	18

**Q6 **How is it decided which participants receive which treatments in the trial?	20 (1)	20	20 (0)	19

**Q11 **Suppose you joined the trial and then decided to drop out. What do the sheets tell you about that?	20 (0)	20	20 (0)	20

**Q12 **What does it say about who might benefit from you taking part in this study?	20 (1)	20	20 (0)	18

**Q14 **Would you have to pay for any of the medicines used?	20 (0)	20	20 (0)	20

**Q15 **Suppose you are harmed by taking part in the trial. What does the sheet say about compensation?	20 (0)	14	20 (0)	18

**Q19 **What does the information sheet say about how the doctors identify which patients should take part in the trial?	20 (1)	19	17 (0)	17

**Trial procedures**				

**Q3 **If you took part in the study, would you have to make any additional visits to the hospital?	19 (1)	19	19 (0)	19

**Q7 **Suppose you took part in the trial and you needed to speak to a doctor urgently one evening. What number should you ring?	20 (1)	20	20 (0)	20

**Q9 **Suppose you took part in the trial and were in the Nafarelin short treatment group. When would you start to use the nasal spray?	20 (0)	20	20 (0)	19

**Q10 **What do the sheets say about taking Progesterone during the trial?	20 (0)	20	19 (1)	19

**Q13 **Suppose you are allocated to be in the Cetrorelix treatment group in the trial. What does it say about how and when Cetrorelix would be given to you?	20 (1)	15	20 (0)	19

**Q16 **Imagine you are in the Nafarelin long treatment group. How much time is there likely to be between starting the nafarelin and having a pregnancy test?	20 (0)	20	20 (0)	20

**Q20 **When taking part in the trial, on each occasion when you have taken your medicine, what would you then need to do?	20 (1)	20	20 (1)	20

**Q21 **Suppose you were taking part and had a concern about the study. How should you contact the doctors in charge?	20 (0)	20	20 (0)	20

**Safety and efficacy of the medicine**				

**Q5 **Please name the two drugs used in the trial that affect the pituitary or reproductive hormones.	20 (0)	20	20 (0)	19

**Q8 **What does the sheet tell you about Follicle Stimulating Hormone or FSH?	20 (0)	9	20 (2)	20

**Q17 **What does the information tell you about getting the side effects of hot flushes and night sweats?	20 (0)	19	20 (0)	20

**Q18 **How does the medicine Cetrorelix work on the body?	20 (0)	20	20 (1)	20

**Total items NOT found (found with difficulty)**	**1(13)/420**		**5(8)/420**	

**Total items NOT understood**		**30/420**		**17/420**

• the nature and purpose of the trial (2 questions);

• the process and meaning of consent (7);

• trial procedures (8);

• safety and efficacy of the tested medicine (4).

We identified the four categories as being those that all clinical trial sheets would contain. The complex nature of IVF treatment meant that the questionnaire in this study featured more questions on trial procedures than were included in the testing of a Phase 1 trial sheet [18]. Conversely, the Phase I sheet testing questionnaire included more items on the safety and efficacy of the trial medicine.

The authors independently selected the 'key points' for questions, based on the pre-defined categories, with any differences reconciled by consensus. The questionnaire was then written, based on the selected items of information. As is normal practice in User Testing, questions were arranged so that their order did not correspond with the order of the information in the sheet. The questionnaire was piloted on 2 participants and we subsequently made minor wording changes. During testing each of the 21 items was scored for *finding *information: *yes*, *no*, or *found with difficulty *(i.e. taking more than 3 minutes) and, if found, scored for *understanding *(*yes *or *no*).

Participants' evaluative comments on the participant information sheets were recorded, as was the time taken to read the information sheet and the time taken for the structured user testing questions.

### Procedure

The study comprised three stages:

(i) *Testing of the original information sheet*. The information was tested using participants who were asked to imagine receiving IVF treatment. They were asked to imagine that they had been asked to take part in a trial to test different ways of giving IVF. They were told that, after having time to read the information through, they would be asked some questions about it. They were left alone to read the sheets and were asked to tell the interviewer when they had finished reading. Then each of the 21 User Test questions was put in turn and the participant was asked, first, to find the answer in the sheet and, second, to give their answer and, where required, to explain what the information meant. No upper time limit was placed on answering each question and the interviewer moved on only if the participant requested or when it became clear that they could not find the answer. After the 21 structured questions participants were asked for their general impressions of the sheet, with particular focus on the appearance, wording, print size, headings and organisation of information. Interviews were audio recorded and transcribed; participants' evaluative comments were analysed according to the headings above.

(ii) *Re-wording and re-design of the Information Sheet*. Revision of the information was based on three sources: participants' User Test questionnaire data and their opinions on the information sheet; best practice in information wording and design [24]; the authors' experience and expertise in information writing and design. Care was taken to retain the original meaning of the information.

(iii) *Testing of the revised Information Sheet *(as per stage (i)), followed by participants' evaluative comments. After completion of the second and third rounds of testing, participants were asked to briefly read a copy of the original sheet and were asked which of the two versions they preferred and why.

### Data analysis

User testing data for the original and revised versions of the sheet were analysed in several ways:

1. We examined the total number of question items that were 'not found' or 'not understood' across the 20 participants who saw each version of the sheet.

2. We identified the number of question items that were found and understood by less than 80% participants (to reflect the threshold recommended in EU legislation on testing of medicine leaflets).

3. We calculated a score for each participant (in the range 0-21) based on how the number of items that each participant could both find and convey an understanding of.

4. We assessed the proportion of participants who were able to find and show an understanding of all 21 question items.

The data for total question items and individual participant scores for the two sheet versions were compared by use of t-tests. The proportion of participants able to find and understand all items were compared by use of the Chi-square statistic.

5. Mean reading and question times were compared for the two versions of the sheet by use of the t-test.

6. Participants' evaluative comments on the sheets were transcribed in full and analysed qualitatively, with themes generated according to common influences on document appraisal, including appearance, layout, wording, use of graphics. We looked for both consensus and contrasts in views, and report these by using illustrative quotes (with the participant's identifying number given in parentheses).

### Research Ethics

Approval to conduct the study was granted by the University of Leeds, School of Healthcare Research Ethics Committee in April 2008.

## Results

### Original information sheet (see Figures 1 and 2)

#### Quantitative data

The original information was tested on two rounds of 10 participants. The data from the first round of ten participants identified some problems with the readability of the information sheet that we wished to confirm with a second round. The 20 women were aged 23-44 (mean 34.3, median 36 years) and ten had personal experience of IVF treatment. Of the twenty participants, nine were higher education graduates, and eight were unemployed or had occupations that did not involve regular use of written documents.

Participants took a mean of 11.9 minutes (range 4 - 22, median 10 minutes) to read the sheet. The period taken to complete the 21 structured questions was a mean of 25.5 minutes (range 14.5 - 45.2 minutes, median 24.4 minutes).

Table 1 shows that participants took a long time to find several answers, as indicated by 'difficulty' ratings (see Table 1). Answers to three of the 21 questions were found and understood by fewer than 80% of the 20 participants. These questions related to:

• FSH, one of the hormones affected by the tested medicines (Q8);

• How and when the tested medicine will be given (Q13); and

• Compensation in case of injury (Q15).

In total 30 (of 420) question items were not understood by participants. When assessing data at a participant level, the 20 participants were able to find and show understanding of a mean of 19.5 items (range 18 - 21). Only 2 of the 20 participants were able to find and show understanding of all 21 question items.

#### Qualitative data

Participants' comments on the sheet were mixed. One positive feature was noted to be the print size, with only 2 of the 20 participants suggesting that it should be larger. For the most part the language was thought to be clear, although some participants struggled to understand the medical terms:

*"We've got a lot of long words here...but I don't think it's too baffling" *(P3);

*"The technical terms are a bit hard to understand" *(P6);

*"It's a bit confusing to work out which drug's having what effect on the body" *(P17).

The organisation of the information was thought to be problematic. Two features mentioned by several participants were the opening page:

*"I'd probably like to know why the study is taking place and who is to benefit on the front or second page" *(P9);

*"There should be more information about what's involved in taking part in the study at the beginning...it launches into quite scientific explanations" *(P13)

and some repetition:

*"The 'do I have to take part?' bit and...'what will happen if you decide to pull out' I don't think is needed to be in twice" *(P11).

The appearance of the sheet was mostly seen positively, including *"professional" *(P8), *"neat and tidy" *(P6), although one participant found the spacing to be *"...a bit random...a bit rushed" *(P9).

When asked for general impressions, comments were split between those who were positive (*"quite thorough" *(P7); and *"informative" *(P16)) and those who found it generally difficult (*"I don't think it was clear" *(P2); *"It does sound a bit confusing" *(P5); and *"it doesn't really make sense" *(P10)).

Others were critical of particular aspects:

*"It's quite confusing to begin with all the FSH, GnRH and so on" *(P16);

*"I found totally confusing the first three pages" *(P3);

*"(the flow diagram) is a little bit misleading" *(P15).

Finally, the name of the trial was criticised, including:

*"It's a strange name for the trial. It makes it sound like you've done something not very well" *(P19), and

*"(the name)...isn't particularly positive" *(P15).

The problems noted in finding and understanding scores, the time taken to answer questions, and participants' comments all indicated that the original information sheet was not performing well. This meant that revision of the information was required, followed by further rounds of testing.

### *Revised information sheet *(see Figures 3 and 4 for examples)

The information sheet was revised and used Linotype Frutiger Next font and presented as a folded booklet of A4 size. Changes to the sheet included:

• Adding to the front page: a brief table of contents and a summary of the most important points;

• Amending the title of the trial;

• Introduction section headings;

• Increased size of page numbers;

• Shortened sentences (where necessary);

• Use of lay language;

• Increasing the font size and removing occasional italics;

• Giving greater emphasis to the contact telephone numbers and placing them on both first and last pages;

• Use of 'bullets' for lists;

• Adding a graphic to illustrate the timing of the various processes, for each of the three trial arms;

• Use of colour for the section headings and the graphic.

The revised information was tested on two rounds of 10 female participants. The data from the first round of 10 participants for the revised sheets showed that it was performing generally well. We tested the sheets on a second round of 10 participants to confirm the data pattern. The 20 participants were aged in the range 23-41 (mean 33.5, median 34.5 years), ten of whom had personal experience of IVF treatment. Nine of the 20 participants were higher education graduates, and eight participants were unemployed or had occupations that did not involve regular use of written documents.

#### Quantitative data

The 20 participants took a mean of 10.4 minutes (range 3-16, median 9.5 minutes) to read the document, a little quicker than for the original document (but the difference is not statistically significant: t = 0.791; p = .44; 95% CIs of the difference -2.48 to 5.48). The period taken for the 21 structured questions was a mean of 20.1 minutes (range 15.7 - 26.0, median 19.5 minutes), a mean reduction of around 5 minutes when compared to the original version (but the difference is not statistically significant: t = 1.875; p = .077; 95% CIs of the difference -0.65 to 11.51).

The problems that participants faced with answering questions 4, 8, 13 and 15 from the original sheet had been rectified. Some minor problems continued, particularly with questions 2 (trial purpose) and 19 (patient identification), but it was felt that there was no easy solution in terms of design or writing. However, all of the answers to questions were found and understood by at least 80% participants. Looking at aggregated data, the revised sheet almost halved the number of items that were not understood by participants: from 30 for the original sheet to 17 for the revised sheet (but the difference is not statistically significant: Chi-square = 3.81; df = 1; p = .051). However, the number of question items not found by participants was 5 (of 420) for the revised sheet, an increase from 1 (of 420) for the original sheet. The difference is not statistically significant (Chi-square = 1.51; p = 0.22; Odds ratio = 0.20; 95% CIs 0.02 to 1.70).

For data at the participant level, the 20 participants were able to find and show understanding of a mean 20.4 items (range 16 - 21), a mean increase of around 1 item when compared with the original sheet. The difference is statistically significant (t = 2.59; p = .019; 95% CIs of the difference 0.17 to 1.63).

Ten of the 20 participants were able to find and show understanding of all 21 question items, a large increase from 2 (of 20) participants for the original sheet version. The difference in proportions is statistically significant (Chi-square = 5.83; p = .006; Odds ratio = 9.0; 95% CIs 1.64 to 49.4).

#### Qualitative data

Participants' comments about the revised sheet were mostly favourable, including its appearance:

*"It doesn't look like it's a scary thing to read" *(P40);

*"It's relatively appealing. I've seen others where it's just block text" *(P26), and

*"It looks...like a leaflet you'd want to pick up and read" *(P37).

The timelines graphics, headings and contents listing were particularly praised:

*"it's nice that there's a graph or timeline as well" *(P32);

*"I answered from there (the timeline) instead of looking in the other stuff. That was really easy to understand in the stages of what went on" *(P33);

*"(The timelines) worked really well because they tell you exactly the stages" *(P27);

*"I think (the headings) are all fine because they are all important questions if anyone was doing the trial they'd want to know. They are all on the contents page at the front" *(P21); and

*"If you look on the front and you need to find out where stuff is you go through each section and I think the headings just sum up what's in it" *(P33).

No participant felt that the print size was too small, and the language used was mostly thought to be at the right level for patients:

*"It's not too technical which is pretty good because a lot of words that are used in IVF can be" *(P27);

*"(There are) no huge big medical words there, they were explained in laymen's terms" *(P28).

However, some participants found the regimen explanations difficult:

*"I found it a bit hard to be honest because I'm not very good at things like that" *(P37).

In general comments, participants found the sheet to be "*really straight forward." *(P32), *"really good...not too over your head or in depth" *(P28), and *"quite self-explanatory" *(P40).

After reviewing the original information sheet, the 20 participants were asked for their preferred version. The revised version was preferred by 18 (90%) participants; one preferred the original and one expressed no preference.

The participant who preferred the original version of the sheet thought that

*"(It) seemed to flow...and there were quite large paragraphs in this (revised version) one" *(P38).

Whereas the participant who could not express a preference said:

*"If I was paying for my own treatment I may prefer (the original version) because I may think that all my money is going to produce glossy pamphlets like (revised version), but in terms of being easier to work your way through (revised version) is better" *(P31).

Among the 18 participants who preferred the revised version, reasons included problems with the original version's layout:

*"The layout of it is a bit poor because it's text heavy on one page" *(P21);

*"It sort of blends into one because you've got the long treatment and the short treatment on the same page you're having to flip over" *(P23);

*"The way it is laid out is very intimidating" *(P26); and

*"It makes me, my brain want to shut down straight away" *(P25).

The title was also not liked:

*"it says 'poor responders' which may make you feel depressed" *(P26);

*"(It's) not a good title to have on the document for people who have been unsuccessful, I don't think that's a very sensitive title to have" *(P30).

By comparison, the revised version was thought to have a better appearance:

*"More professional...more like a patient information leaflet you'd pick up in hospitals" *(P22);

*"It makes you feel like it's not just come from a printer, it's been produced with a lot of thought and care" *(P24);

*"The look, it seems clear, each heading in bold print so you know where to look for certain points...the fact that it's got colour helps" *(P36); and

*"You're attracted to read it from cover to cover" *(P40).

The appearance of the revised sheet was also thought to have an impact on the appeal and readability of the information the sheet contains:

*"Possibly it seems like there is less information in (revised version) but I think actually it gives you more. Because of the way it's laid out you pick up more" *(P22); and

*"It looks easier to follow and understand. The writing's spread out more. This (original version) looks like too much information, this (revised version) doesn't." *(P35).

## Discussion

Performance-based readability testing of the participant information sheet for the 'Poor Responders' trial showed that it performed sub-optimally. Members of the public took a long time to find some aspects of the trial. When information was found, it was not always understood. Three important aspects of the trial were not understood by five or more of the 20 participants. Revising the document - by re-writing and re-designing it, while retaining its meaning - led to improvements in its performance. In particular, almost all the found information was understood correctly.

The original sheet performed well in some places and participants were able to find and understand an average of 19.5 items (not far below the 'ceiling' of 21 items). Sheet revisions increased the mean to more than 20. Sheets revisions led to a fall in the number of 'not understood' items, but an increase in the number that were 'not found', showing a mixed effect of sheet revision. From a clinical perspective, however, it is perhaps the increase in the proportion of participants able to find and understand all items that is most meaningful: this figure jumped from 10% to 50%.

In general, the results of this study support the views of Ancker [17] and others that the analysis of the trial Participant Information Sheet needs to be based on its performance, rather than on a number obtained from a readability formula. For, at worst, documents can be manipulated to perform better on readability formulae, with little or any real change in the ease with which participants can use the information. Readability formulae can give an indication of the usability of a document, but they offer very limited value in terms of indicating which aspects of a document 'work' and which do not. An alternative approach would be to use a less structured method, with a focus on people's opinions on a document. However, it is not clear how closely people's opinions on a document match its ability to inform, and we are aware of no studies that make such a link.

As outlined previously, the sheet was revised with reference to three sources: best practice in information design and writing; authors' expertise; and the data obtained from the test of the original version. One pertinent question is whether testing data are necessary, or whether a document can be revised from expert knowledge and best practice alone. A recent review of information design in relation to medicines information [27] argues that the discipline is more than just a set of principles or rules for good design; an analysis of the needs of information users is also essential. The collection of testing data allows such needs to be indicated. Published research also supports the view that testing data are needed: experts are not good at predicting the problems that readers will face when trying to read and understand a document [28]. The revision of the trial information in this study did make explicit reference to the participant data obtained; for example, the inclusion of a revised timeline, an amended title, and re-worded explanation of the drugs and hormones relevant to IVF.

There are six questions (numbers 4, 5, 6, 10, 12 and 19; see Table 1) where proportionally more people could not find or understand information in the revised sheet than in the original version. The increase in 'not found' items is not statistically significant, and the differences may be due to chance or to variation in participants between the 'original' and 'revised' groups. We sought to match the groups, and did so successfully, based on several relevant patient characteristics. In the authors' experience it is unusual that a document performs in User Testing such that all items are 'found' and 'understood' by all participants - in this study we were able to achieve 50% participants doing so. The sheet for the 'Poor Responders' trial might have been further revised and tested on additional rounds of participants, although it is questionable whether further improvements in performance would have resulted. Rather, the interpretation of User Testing data should probably emphasise the pattern of scores obtained across the questionnaire, and on this basis the revised information performed well.

The participants in this study were relatively well educated: the proportion of higher education graduates was much higher than in the general population, for instance. In part this is due to our aim that half the participants had personal experience of IVF: these participants were recruited via support groups that tended to have more highly educated members. It would be important that future studies of trial participant information sheets include samples that more closely resemble the characteristics of actual trial participants.

The User Testing scores obtained for the original trial sheet would question whether a participant in this trial would have been fully informed before giving consent, particularly since just 10% participants could find and understand answers to all questions. Furthermore, participants would not have been able to use the sheet easily as a resource, after giving consent and when away from the hospital. This is important since research suggests that verbal information alone will not be enough for many patients; for example, most people quickly forget a third to a half of the information they are given in a consultation [29,30].

The tested sheet was not particularly lengthy, certainly not by comparison with many trial sheets, particularly those used in Phase 1 trials. However, it did include technical and complex information - both on the medicines and the effect on the body, and the process and timing of using the medicines. In testing of the original sheet even those participants with personal IVF experience were among those unable to answer questions 8 (on Follicle Stimulating Hormone) and 13 (on dosage timing) correctly. Revision of the sheet's wording and the addition of a graphic resulted in this complex information being understood: all 20 participants, including the 10 participants with no personal experience of IVF, were able to understand the information to answer those two questions.

Further evidence from medicine prescribing outside trials is that patients prefer to receive information from clinicians in a spoken form; but they want written information to complement it [31]. As stated earlier, the participant information sheet could be seen to perform several functions: pre-consent it informs and provokes questions; post-consent it acts as a memory aid and a record for the participant. The aspiration of health information design and writing is that any document intended for the patient should be good enough for them to read and understand without the need to seek clarification. The User Testing method evaluates the performance of written information under such circumstances. Consent to clinical research, including trials, is based on a combination of written information in the form of a participant information sheet and spoken information from a clinician. The data in this study indicate the difficulty faced in writing complex trial information such that participants can understand it all: the complementary spoken information from a clinician appears to be essential to clarify and explain.

The User Testing method uses small numbers of participants and so cannot give the statistical certainty of larger quantitative research designs. Rather, it is a detailed and iterative process, well established in the Information Design domain [19]. When used in practice the main deficiencies in a document can be identified after interviewing just 10 potential users. However, it would require a study comparing the original and revised versions, with random allocation of participants, to confirm the pattern of scores reported here. The process of revising the sheet by using data from the testing of the original version, meant that it was not possible to conduct the study with random allocation to parallel groups. Larger scale research might include sub-group analyses in order to answer important questions including whether less educated participants gain more benefit from the revision of information sheets and, pertinent to this population, whether participants with experience of IVF do better. The small sample recruited here was appropriate for the study aims, since User Testing can reveal most of the problems associated with a document when tested on rounds of 10 or 20 participants [19].

It is important to note that this study does not address the appropriateness of the content of the original participant information sheet: it simply shows that the wording and layout of the original information were sub-optimal in terms of patient understanding.

This study suggests that the materials provided for participants in this trial might fail to inform them fully. The 'Poor Responders' trial information was not particularly lengthy (sheets often run to 15 pages or more), and longer trial sheets may well perform less well than this. We need further research to indicate whether other examples of trial information can be understood by potential participants.

## Conclusion

User Testing can allow information to be evaluated in a structured way. When it is combined with expertise in writing for patients and information design, it may result in a greater proportion of patients being able to understand what will happen within a trial. Not only would this impact on the extent to which valid consent is given, but it may also impact on the smooth running of the trial, with participants knowing where and when to take medicines, return for tests, etc. Increasingly potential participants have been involved in the development of trial materials, often resulting in the materials being altered substantively [32]. The great strength of performance-based testing is that it allows confirmation (or otherwise) that such changes will be of benefit to participants.

## Competing interests

D K Raynor is a director of Luto Research Ltd, a University of Leeds spin-out company that provides information writing and testing services to the pharmaceutical industry. Jonathan Silcock is chair of an NHS Research Ethics Committee, but the views expressed are those of the authors not necessarily those of the National Research Ethics Service. Brian Parkinson provides graphic design services to the pharmaceutical industry and the NHS.

## Authors' contributions

PK and DKR conceived the study idea. PK, DKR and JS designed the study method and wrote the revised participant information sheet. BP designed the revised information sheet. PK managed the data collection and analysis. All authors contributed to writing the article.

## References

[B1] Griffin JM, Struve JK, Collins D, Liu A, Nelson DB, Bloomfield HE (2006). Long term clinical trials: how much information do participants retain from the informed consent process?. Contemporary Clinical Trials.

[B2] Kaewpoonsri N, Okanurak K, Kitayaporn D, Kaewkungwal J, Vijaykadga S, Thamaree S (2006). Factors related to volunteer comprehension of informed consent for a clinical trail. Southeast Asian Journal of Tropical Medicine and Public Health.

[B3] Lynoe N, Saundland M, Dahqvist G, Jacobsson L (1991). Informed consent: study of quality of information given to participants in a clinical trial. British Medical Journal.

[B4] Fortun P, West J, Chalkley L, Shonde A, Hawkey C (2008). Recall of informed consent information by healthy volunteers in clinical trials. Quarterly Journal of Medicine.

[B5] Cox AC, Fallowfield LJ, Jenkins VA (2006). Communication and informed consent in phase 1 trials: a review of the literature. Support Care Cancer.

[B6] Royal Statistical Society (2007). Report of the Working Party on Statistical Issues in First-in-Man studies.

[B7] Jenkins VA, Fallowfield LJ, Souhami A, Sawtell M (1999). How do doctors explain randomised clinical trials to their patients?. European Journal of Cancer.

[B8] Sugarman J, McCrory DC, Hubal RC (1998). Getting meaningful informed consent from older adults: a structured literature review of empirical research. Journal of the American Geriatric Society.

[B9] Stryker JE, Wray RJ, Emmons KM, Winer E, Demetri G (2006). Understanding the decisions of cancer clinical trial participants to enter research studies: Factors associated with informed consent, patient satisfaction, and decisional regret. Patient Education and Counseling.

[B10] Expert Scientific Group on Phase One Clinical Trials (2006). Final Report.

[B11] Flesch R (1948). A new readability yardstick. Journal of Applied Psychology.

[B12] Kincaid JP, Fishburne RP, Rogers RL, Chissom BS (1975). Derivation of new readability formulas (Automated Readability Index, Fog Count and Flesch Reading Ease Formula) for Navy enlisted personnel.

[B13] McLaughlin GH (1969). SMOG Grading - a new readability formula. Journal of Reading.

[B14] Paasche-Orlow MK, Taylor HA, Brancati FL (2003). Readability standards for informed-consent forms as compared with actual readability. New England Journal of Medicine.

[B15] Burman W, Breese P, Weis S (2003). The effects of local review on informed consent documents from a multicenter clinical trials consortium. Controlled Clinical Trials.

[B16] Mayo DJ (2003). Readability of Informed-Consent forms. New England Journal of Medicine.

[B17] Ancker J (2004). Assessing patient comprehension of informed consent forms. Controlled Clinical Trials.

[B18] Knapp P, Raynor DK, Silcock J, Parkinson B (2009). Performance-based readability testing of participant materials for a Phase 1 trial: TGN1412. Journal of Medical Ethics.

[B19] Sless D, Wiseman R (1997). Writing about medicines for people.

[B20] European Commission Draft Guideline on the readability of the label and package leaflet of medicinal products for human use. Revision September 2006. http://ec.europa.eu/enterprise/pharmaceuticals/pharmacos/docs/doc2006/09_2006/readability_consultation_2006_09_25.pdf.

[B21] Medicines and Healthcare products Regulatory Agency (2005). Always read the leaflet: Getting the best information with every medicine.

[B22] Raynor DK, Svarstad B, Knapp P, Aslani P, Rogers MB, Koo M, Krass I, Silcock J (2007). Consumer Medicines Information in the United States, Europe and Australia - a comparative evaluation. Journal of the American Pharmacists Association.

[B23] Dresden GM, Levitt MA (2001). Modifying a standard industry clinical trial consent form improves patient information retention as part of the informed consent process. Academic Emergency Medicine.

[B24] Coyne CA, Xu R, Raich P, Plomer K, Dignan M, Wenzel LB, Fairclough D, Habermann T, Schnell L, Quella S, Cella D, Eastern Cooperative Oncology Group (2003). Randomized, controlled trial of an easy-to-read informed consent statement for clinical trial participation: a study of the Eastern Cooperative Oncology Group. Journal of Clinical Oncology.

[B25] Robinson EJ, Stevens AJ, Lilford RJ, Braunholtz DA, Edwards SJ, Beck SR, Rowley MG (2005). Lay public's understanding of equipoise and randomisation in randomised controlled trials. Health Technology Assessment.

[B26] Sunkara SK, Coomarasamy A, Khalaf Y, Braude P (2007). A three-arm randomised controlled trial comparing Gonadotrophin Releasing Hormone (GnRH) agonist long regimen versus GnRH agonist short regimen versus GnRH antagonist regimen in women with a history of poor ovarian response undergoing in vitro fertilisation treatment: Poor responders intervention trial (PRINT). Reproductive Health Journal.

[B27] Raynor DK, Dickinson D (2009). Key principles to guide development of consumer medicines information - content analysis of information design texts. Annals of Pharmacotherapy.

[B28] Lentz L, De Jong M (1997). The evaluation of text quality: expert-focused and reader-focused methods compared. IEEE Transactions on Professional Communication.

[B29] Ley P (1973). Communication in the clinical setting. British Journal of Orthodontics.

[B30] Wilson M, Robinson EJ, Blenkinsopp A, Panton R (1992). Customers' recall of information given in community pharmacies. International Journal of Pharmacy Practice.

[B31] Raynor DK, Blenkinsopp A, Knapp P, Grime J, Nicolson DJ, Pollock K, Dorer G, Gilbody SM, Dickinson D, Maule AJ, Spoor PA (2007). A systematic review of quantitative and qualitative research on the role and effectiveness of written information available to patients about individual medicines. Health Technology Assessment.

[B32] Koops L, Lindley RI (2002). Thrombolysis for acute ischaemic stroke: consumer involvement in design of new randomised controlled trial. British Medical Journal.

